# Mobitz type I as manifestation of acute lithium cardiotoxicity

**DOI:** 10.1016/j.toxrep.2023.05.004

**Published:** 2023-05-17

**Authors:** Henrik Galust, Justin Seltzer, Jeremy Hardin, Nate Friedman, Alicia Minns

**Affiliations:** UCSD Fellowship in Medical Toxicology, Department of Emergency Medicine,UCSD Medical Center, 200 W. Arbor Dr. #8676, San Diego, CA 92103, United States

**Keywords:** Lithium, Cardiotoxicity, Mobitz type I, Wenckebach, EKG

## Abstract

Lithium induced cardiotoxicity is associated with several electrocardiographic (ECG) findings. The most commonly observed cardiac effects include QT prolongation, Twave abnormalities, and to lesser extent SA node dysfunction and ventricular arrythmias. We present a case of a 13-year-old female with acute lithium overdose whodeveloped Mobitz I, a manifestation of lithium associated cardiotoxity not previously reported. The patient had no significant past medical history and presented to the emergency department 1 h after intentional overdose of 10 tablets of unknown drug. Parents reported that the patient had visited her grandmother, who “regularly took many different kinds of medications,” earlier that same evening. On physical examination the patient had reassuring vital signs, was in no acute distress,cardiopulmonary examination was normal, had clear sensorium, and no evidence of a toxidrome. On serological examination complete blood count, chemistries panel, and liver function tests did not show significant derangements. 4 h post-ingestion acetaminophen concentration was 28 mcg/ml and below indication for n-acetylcysteine antidote therapy. During her ED course she showed evidence of Mobitz I (Wenckebach) on 12-lead ECG. No prior ECGs were available for comparison. Medical toxicology was consulted at that time given concern for potential cardiotoxicity from an unknown xenobiotic. Serum dioxin and lithium concentrations were subsequently requested. Serum digoxin concentration was undetectable. Serum lithium concentrations was 1.7 mEq/L (0.6–1.2 mEq/L therapeutic range). The patient was treated with intravenous hydration at twice maintenance rate. Repeat lithium concertation 14 h post-ingestion was undetectable. During her admission, the patient remained hemodynamically stable and asymptomatic despite occasional episodes of Mobitz I, lasting seconds to minutes. Repeat 12-lead ECG obtained 20 h post-ingestion showed normal sinus rhythm. Cardiology recommendations included ambulatory Holter monitoring upon discharge and follow-up in clinic within two weeks. The patient was medically cleared after 36 h of monitoring and discharged after psychiatric evaluation. Our case demonstrates that patients who develop a new Mobitz I atrioventricular block of unclear etiology in the setting of acute ingestion should be screened for lithium exposure, even if otherwise free of more typical manifestations of lithium toxicity

## Highlights

• Lithium associated neurotoxicity is more common than cardiotoxicity.
• Cardiotoxicity can present with T-wave abnormalities, QT interval prolongation, sinus node dysfunction, and ventricular arrhythmia.
• Patients with a new Mobitz I atrioventricular block of unclear etiology in the setting of acute ingestion should be screened for lithium exposure.

## Graphical Abstract

**Figure:**
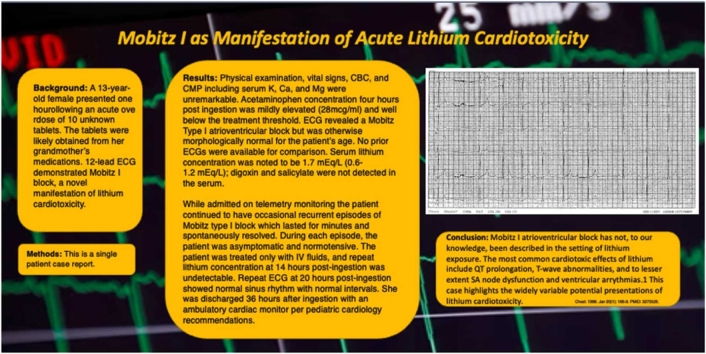

## 1 Introduction

Lithium is one of the most popular treatment options for acute mania and maintenance therapy for bipolar disorder [1]. However, it has a narrow therapeutic index and is a common reported cause of psychoactive medication toxicity [2]. Lithium is known to cause cardiotoxicity, specifically sinus node dysfunction [3], [4], [5], [6], [7], [8]. However, a Mobitz I atrioventricular block has, to our knowledge, not been reported in a pediatric patent.

Here we present a case of Mobitz type 1 atrioventricular block in a 13 year-old patient who presented after an intentional lithium ingestion.

## 2 Case report

A 13 year-old healthy female presented approximately 1 h after an intentional overdose of 10 tablets of an unknown drug. She was asymptomatic at the time of presentation. Parents reported acetaminophen, aspirin, fluoxetine, trazadone, and prazosin were available in the home and reported that the patient had also visited her grandmother earlier that same evening. It was unknown at that time what medications the grandmother was prescribed.

The patient’s vital signs were: blood pressure 128/87 mmHg, heart rate 82 beats per minute, respiratory rate 16 breaths per minute, peripheral oxygen saturation 99% on room air, and temperature 37.4 °C. The initial physical examination revealed no abnormalities. No hyperreflexia, tremor, or clonus were noted. A complete blood count and comprehensive metabolic panel were within normal limits. The four hour acetaminophen concentration was 28 µg/ml. Serum aspirin and ethanol concentrations were undetectable. Urine drugs of abuse screening was negative for amphetamine, barbiturates, benzodiazepines, cannabinoids, cocaine, opiates, and PCP.

**Fig. 1:**
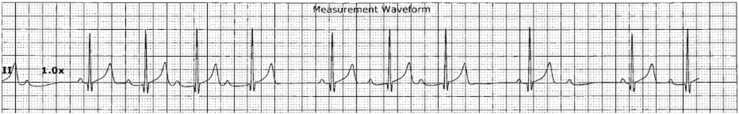
Irregular cardiac activity was noted on telemetry.

**Fig. 2:**
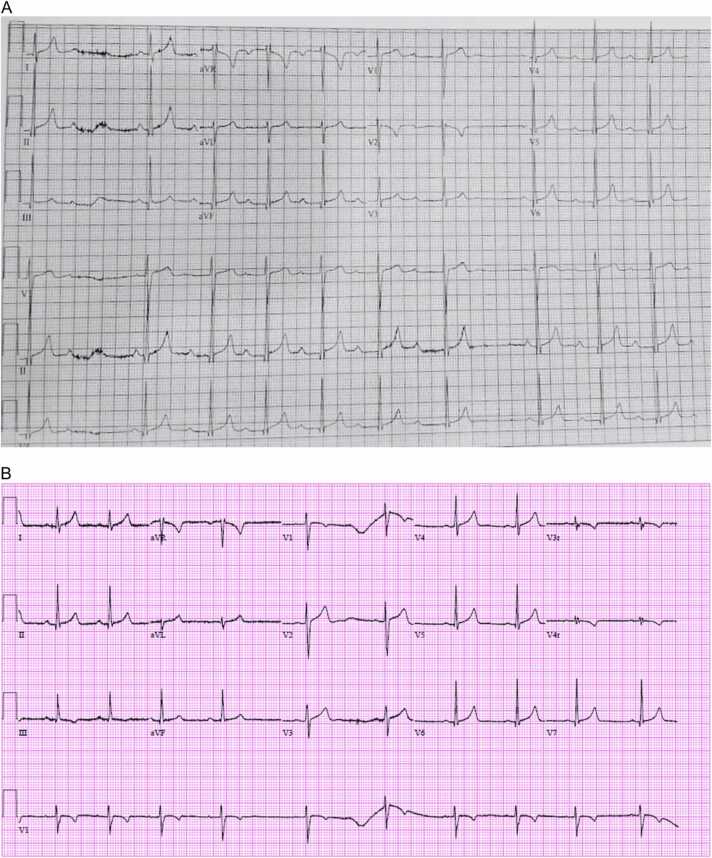
A. 12-lead EKG with evidence of Mobitz I (Wenckebach) atrioventricular block.B. 12-lead EKG demonstrating sinus arrhythmia.

After the patient’s initial medical examination, she was observed overnight on telemetry pending psychiatric evaluation in the morning. Approximately 10 h after ingestion, irregular cardiac activity was noted on telemetry (Fig. 1). A 12-lead ECG (Fig. 2A) was obtained to further characterize this rhythm, which was determined to be a Mobitz I (Wenckebach) atrioventricular block. No prior ECGs could be obtained for comparison. Vital signs remained normal during this time and the patient continued to be asymptomatic. The toxicology service was consulted at that time given concern for potential cardiotoxicity from her unknown ingestion.

Toxicology recommendations included obtaining serum digoxin and lithium concentrations from index lab draw. Serum digoxin concentration was undetectable. Serum lithium concentration was 1.7 mEq/L (laboratory therapeutic range: 0.6–1.2 mEq/L). The patient was treated with intravenous fluids administered at twice the maintenance infusion rate for 14 h, after which a repeat lithium concentration was obtained and found to be less than the lower limit of detection. A repeat 12-lead EKG obtained approximately 20 h post-ingestion showed normal sinus rhythm with sinus arrhythmia (Fig. 2B).

The patient was medically cleared after 36 h of monitoring and, following psychiatry clearance, discharged with an ambulatory cardiac monitor and cardiology clinic follow up.

## 3 Discussion

Lithium is primarily neurotoxic, commonly causing tremor, confusion, ataxia, and manifestations of hyperreflexia. Patients chronically taking lithium generally experience more serious toxicity compared with lithium naïve patients suffering acute ingestions [3], [4]. Cardiotoxicity occurs as well, though it is less common than neurotoxicity [5]. T-wave abnormalities, QT interval prolongation, sinus node dysfunction, and ventricular arrythmias are all well reported [6]. However, to our knowledge, Mobitz I (Wenckebach) atrioventricular block specifically has not been described in the setting of lithium toxicity.

Lithium cardiotoxicity is thought to be due to direct ion substitutions in transport processes, which leads to inhibition of cardiac voltage-gated sodium channels and decreased intracellular potassium. Consequently, lithium decreases spontaneous depolarization and impedes conduction of the atrioventricular node and ventricles [7], [8]. These electrolyte substitutions likely account for the most commonly observed manifestations of QT interval prolongation and T-wave inversion or flattening [9], [10].

This case has three major limitations. First, we cannot rule out the possibility of a baseline Mobitz I atrioventricular block as the patient had never had telemetry monitoring or an ECG performed previously. Second, although the patient had a detectable acetaminophen and lithium concentration, we cannot rule out other ingestions. Finally, as lithium was not known to be a potential ingestion, we acknowledge that the detected serum lithium concentration was possibly erroneous. Factitiously elevated serum lithium concentrations have been reported when blood samples were analyzed containing the anticoagulant lithium-heparin [11], [12] We did confirm with our laboratory that the patient’s initial blood samples did not contain lithium heparin, however it remains possible that sample error or contamination caused the detected lithium concentration.

## 4 Conclusion

We report an otherwise asymptomatic patient who developed a Mobitz I atrioventricular block in the setting of an elevated serum lithium concentration. We conclude that patients who develop a new Mobitz I atrioventricular block of unclear etiology in the setting of acute ingestion should be screened for lithium exposure, even if otherwise free of more typical manifestations of lithium toxicity.

## Declaration of Competing Interest

The authors declare that they have no known competing financial interests or personal relationships that could have appeared to influence the work reported in this paper.

## Data Availability

No data was used for the research described in the article.
